# Dietary Patterns and Factors Associated with Food Affinity in Pregnant Women from Quito, Ecuador

**DOI:** 10.3390/nu16040475

**Published:** 2024-02-07

**Authors:** Paola Toapanta-Pinta, Santiago Vasco-Morales, Sara Céspedes-Granda, Daniela Saes Sartorelli, Elaine Christine Dantas Moisés

**Affiliations:** 1 Obstetrics Career, Universidad Central del Ecuador, Quito 170403, Ecuador; snvasco@uce.edu.ec (S.V.-M.); scespesdes@uce.edu.ec (S.C.-G.); 2 Ribeirão Preto Medical School, University of São Paulo, Ribeirão Preto 14040-900, Brazil; 3 Neonatology Department, Hospital Gineco-Obstétrico Isidro Ayora, Quito 170136, Ecuador; 4 Puengasí 2 Health Center, District 17D04 Puengasí in Itchimbia, Quito 170145, Ecuador; 5 Department of Social Medicine, Ribeirão Preto Medical School, University of São Paulo, Ribeirão Preto 14040-900, Brazil; daniss@fmrp.usp.br; 6 Department of Gynecology and Obstetrics, Ribeirão Preto Medical School, University of São Paulo, Ribeirão Preto 14049-900, Brazil; elainemoises@fmrp.usp.br

**Keywords:** nutritional behaviour, pregnancy nutrition, pregnant women, diet habits, dietary patterns

## Abstract

Nutrition during pregnancy influences perinatal outcomes and predispositions to chronic diseases. A prospective cohort study was carried out with the objectives of describing the dietary patterns in the pregnant population in the city of Quito, Ecuador and analysing the sociodemographic and lifestyle factors that influence the adherence to each dietary pattern. The body mass index was calculated for each patient, and the patients were classified according to the Atalah criteria. The Global Physical Activity Questionnaire was also applied. The dietary patterns were assessed using a dietary survey with a 24 h recall on two occasions. A total of 535 pregnant women were included. A positive association was found between the pattern “dairy, salads and sweet snacks/dressings” and foreign nationality (β = 0.82 (0.43;1.21)). The “refined carbohydrates” pattern was negatively associated with education equal to or less than 7 years and an income of up to one basic salary (β = −0.59 (−1.05; −0.14)). The “traditional Ecuadorian” pattern showed a positive association with being born in the coastal region of Ecuador (β = 0.62 (0.22; 1.01)). This study identified three dietary patterns in pregnant women and their possible associations with certain sociodemographic factors. More studies are needed to better understand these patterns as well as to analyse their nutritional and caloric properties.

## 1. Introduction

Maternal nutrition during pregnancy is a key factor in ensuring adequate foetal growth and development as well as in preventing short- and long-term complications in both the mother and the newborn [1].

According to the World Health Organization (WHO), adequate maternal nutrition during pregnancy remains an objective to achieve. The WHO recommends that pregnant women’s diets provide sufficient energy, protein, vitamins, and minerals from a variety of foods, including vegetables, lean meats, fish, legumes, whole grains, and fresh and dried fruits [2].

Assessing dietary patterns during pregnancy will allow us to (1) identify the main sources of nutrients in Ecuadorian pregnant women; (2) identify deficiencies or nutritional excesses related to sociodemographic factors; (3) design culturally sensitive strategies to promote healthy eating tailored to each region; (4) prevent maternal diseases such as gestational diabetes and hypertension, which, in turn, impact foetal growth; (5) reduce the risk of foetal growth disorders such as macrosomia or intrauterine growth restriction; and (6) decrease the development of chronic diseases in offspring in the long term [3,4,5].

The assessment of dietary patterns reflects how foods are consumed together and allows us to understand how they interact with social, demographic, and lifestyle factors during gestation. These findings also allow for the establishment of specific public health interventions for each population aimed at promoting affordable and healthy diets to improve maternal and child health [6,7].

Due to its geographical location, Ecuador is divided into four geographic regions, namely the highlands, the coast, the east, and the insular zone, which contributes to a wide food biodiversity. These geographical conditions favour the cultivation and harvesting of a variety of foods throughout the year, enriching its food supply [8].

Nutritional studies in Ecuador have led to the creation of photographic food atlases, providing data on their calorie or gram equivalents. However, as far as we know, specific dietary patterns have not been established for the country’s various population groups [9,10].

The aim of this study was to describe the dietary patterns of the pregnant population in the city of Quito, Ecuador and to analyse the sociodemographic and lifestyle factors influencing the adherence to each dietary pattern.

## 2. Materials and Methods

### 2.1. Type of Study and Population

A prospective cohort study was conducted that included 535 pregnant women selected among individuals attending obstetric consultations in the city of Quito, Ecuador from January 2021 to November 2022. The patients were recruited during an obstetric consultation in Health District 17D04 Puengasí to Itchimbia, whose coverage area corresponds to the urban areas of northern central Quito and the Isidro Ayora Gynecological-Obstetric Hospital, which is a third-level national reference hospital. During the first prenatal visit, pregnant individuals were invited to participate in the study, and the inclusion criteria were as follows: aged between 19 and 39 years and a gestational period of up to 14 weeks. Women with diabetes mellitus and/or arterial hypertension, as well as those using medications affecting glucose metabolism, were excluded.

Quito is the capital of Ecuador and is located 2830 metres above sea level in the Andes Mountain range; it covers 4183 km^2^ and has a temperature range of 10–25 °C. With a population of approximately 1,911,966 people, the number of women is 988,486 (51.7%), and 53.2% of them are of reproductive age; migration, mainly from Venezuela, constantly influences these figures [11].

The sample size was calculated using the Quantitative Methods Center (CEMEQ) calculator [12] considering the following parameters: an incidence of gestational diabetes mellitus (GDM) in the exposed group (obesity) of 0.27; an incidence of GDM in the nonexposed group (non-obesity) of 0.17; a significance level of 5%; and a confidence level of 80%. The result yielded a sample size of 532 pregnant women.

In terms of frequency, it was observed that, on average, the obstetric consultation in District 17D04 Puengasí to Itchimbia attended to 2440 pregnant individuals every year, while the outpatient consultation at the HGOIA had an average annual attendance of 2114 pregnant individuals. Of the women included in this study, 208 (38.3%) received care from the HGOIA and 327 (61.1%) received care from District 17D04 Puengasí to Itchimbia.

### 2.2. Data Collection

**Figure 1:**
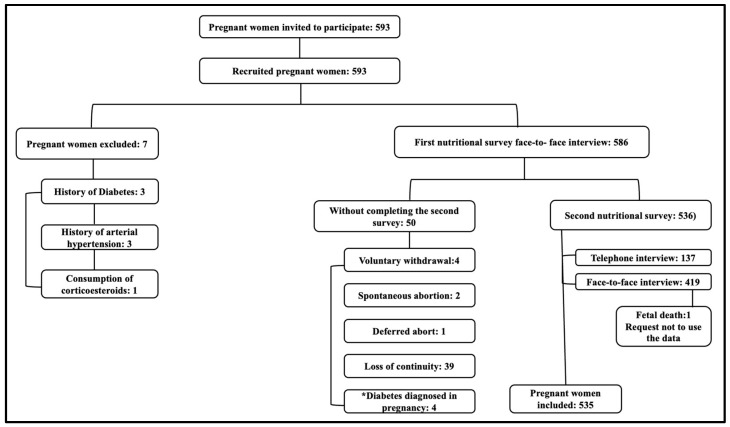
Flowchart of study participant selection. * Initial blood glucose ≥ 126 mg/dL.

A total of 629 pregnant women who met the inclusion criteria were invited to participate in the study, of whom 36 decided not to participate. Another 7 patients were excluded during the collection of data on sociodemographic characteristics and personal medical history, and 51 pregnant women experienced discontinuity of care during the study. The analysis included a total of 535 pregnant women. The study flowchart is presented in Figure 1, illustrating the study’s approach.

Sociodemographic and lifestyle information was collected through a structured questionnaire. The sociodemographic variables included age (up to 20 years old, 20 to 35 years old, or over 35 years old), nationality (Ecuadorian or foreign), region of origin in Ecuador (highlands, coast, eastern region, or Galapagos Islands), level of education (up to 7 years, 8 to 13 years, or 14 years or more), marital status (living with a partner or not living with a partner), self-identified ethnicity (mestizo, indigenous, Afro-descendant, or other), occupation status (paid employment or unpaid employment), and income based on Ecuador’s basic salary (up to 1 basic salary equivalent (USD 400), 1–2 basic salary equivalents (USD 800), 2–3 basic salary equivalents (USD 1200), or more than 3 basic salary equivalents (more than USD 1200)).

Additionally, the following variables were included: personal medical history (yes or no); family history of metabolic diseases such as diabetes mellitus, obesity, dyslipidaemia, or hypertension (yes or no); history of previous pregnancies (yes or no); number of previous pregnancies (1 to 3, 4 or more); history of previous miscarriages (yes or no); history of previous deliveries (yes or no); and history of previous caesarean sections (yes or no).

The following lifestyle variables were included: history of smoking (used to smoke, currently smoking, or exposed to second-hand smoke) and alcohol consumption (used to consume or currently consuming).

### 2.3. Assessment of Physical Activity Level

The level of physical activity was determined using the Global Physical Activity Questionnaire (GPAQ) in Spanish [13] following the instructions provided in its analysis and interpretation guide. The questionnaire assesses the following areas: work, commuting, and leisure time for a typical day in a typical week. The intensity of activity was classified as moderate and vigorous for work and leisure time and only as moderate for commuting [14]. 

To calculate the categorical indicators, the total time dedicated to physical activity during a typical week and the intensity of the physical activity were considered, where the time and intensity of the physical activity were transformed into metabolic equivalents (METs) to calculate the total physical activity. An MET is the ratio of a person’s working metabolic rate to their resting metabolic rate, and it is defined as the energy cost of sitting quietly and is equivalent to a caloric consumption of 1 kcal/kg/h. It was estimated that the caloric expenditure of a person was four times higher when moderately active and eight times higher when vigorously active compared to when sitting. Therefore, when calculating the total energy expenditure of a person using the GPAQ data, 4 METs were assigned to the time spent performing moderate physical activities, and 8 METs were assigned to the time spent performing vigorous physical activities. According to the classification of the WHO, to be considered active, individuals should perform either 150 min of moderate physical activity or 75 min of vigorous physical activity, or a combination of both moderate and vigorous activities in equivalent proportions, achieving a total of at least 600 METs/minute. Therefore, pregnant women were classified as physically active when it was determined that they achieved 600 METs/minute or more, and they were classified as inactive when they achieved equivalent to or less than 600 METs/minute. The GPAQ was also used to estimate sedentary time; therefore, the sedentary behaviour of pregnant women was classified based on time: up to 4 h and more than 4 h [14].

### 2.4. Calculation of Body Mass Index (BMI)

Weight data in kilograms (kg) and height data in centimetres (cm) were obtained from the perinatal records. A mechanical DETECTO brand scale was used to measure the weights and heights of pregnant women in institutions belonging to the Ministry of Public Health of Ecuador, which consisted of a weighing beam and a height measure. Subsequently, the calculation of the body mass index (BMI) was performed. In accordance with the prenatal control guidelines of the Ministry of Public Health of Ecuador [15], the criteria proposed by Atalah (1997) were applied. These criteria establish that the weight up to the 13th week of pregnancy can serve as a reference for the pregestational weight. Based on these parameters, the BMI adequacy was classified, according to the gestational week, into the categories of underweight (<18.5), normal weight (18.5 to 24.9), overweight (25 to 29.9), and obesity (≥30) [16]. 

### 2.5. Assessment of Eating Habits and Dietary Patterns

To determine the participants’ dietary habits, the food consumption assessment technique was used in two 24 h dietary recalls (IR24HR). For data collection, the following steps were taken: (1) a quick list of foods consumed on the previous day was made; (2) data about forgotten foods were grouped into categories such as beverages, sweets, snacks, fruits, vegetables, and cheeses; (3) information was collected about the time and location of consumption, indicating the occasion as breakfast, lunch, snack, etc.; (4) each food/drink consumed was described, along with its quantity, and the timings and occasions were reviewed to check if any food had been omitted; and (5) a final review was performed to include additional foods that were not previously recalled [17,18].

The first 24 h dietary recall (IR24HR) was performed at the time of recruitment through a face-to-face interview, and the second was conducted through a face-to-face interview or by telephone 7 days to 1 month after the first survey. To estimate the food portions, the photographic manual for food quantification from Ecuador’s San Francisco de Quito University was used [9]. Because diet can vary greatly from one day to another and it is not appropriate to use information from a single 24 h dietary recall to characterise a person’s habitual diet [17], the foods mentioned in the 24 h dietary recalls of the pregnant women who completed both surveys were grouped into 16 food groups based on their nutritional values or consumption logic.

The Multiple Source Method (MSM), which is a statistical modelling technique that utilises the distribution of habitually consumed foods obtained through a 24 h dietary recall, was used to determine the usual consumption of food groups by pregnant women. The MSM employs a three-step technique to estimate habitual nutrient and food intake. First, the probability of a nutrient being consumed by an individual is calculated based on the foods consumed on a random day. Second, the usual amount of intake by the individual is estimated. Finally, the probability of consumption is multiplied by the usual intake amount to obtain the habitual intake. This approach allows for the variability in intake to be adjusted without requiring many repeated dietary surveys [19].

To identify dietary patterns, the principal component analysis method was used in an exploratory factor analysis (EFA), which allowed for the association of food groups based on their degree of correlation. This approach helped to identify dietary patterns and potential relationships among different food groups. Negative values in the EFA indicated an inverse association of the variable with the factor, while positive values indicated a direct association [18].

To identify the number of patterns to retain in the EFA, a cut-off of eigenvalues greater than 1.5 was used. The R (version 4.1.2) software indicated that retaining three dietary patterns was sufficient. After applying the orthogonal Varimax rotation, food groups with a factor loading greater than 0.25 were retained. This indicated that these food groups had a strong contribution to the pattern identified in the EFA. The factorial scores in the dietary pattern of each pregnant woman were calculated using the Bartlett method. Subsequently, the affinity of each pregnant woman for each dietary pattern was determined by classifying their scores into tertiles. Women with scores in the first, second, and third tertiles were considered to have low, medium, and high affinities for the dietary patterns, respectively [18,20].

### 2.6. Data Analysis

For the data analysis, the absolute and relative frequencies of the sociodemographic and lifestyle variables of the pregnant women were calculated. The proportions of sociodemographic and lifestyle characteristics were determined concerning the affinity or adherence of the pregnant women to each dietary pattern using the chi-squared test. Linear regression models were used to explore the relationships between sociodemographic and lifestyle variables and dietary pattern scores. Following Gomes’ methodology, all variables with association levels of *p* < 0.20 in the bivariate analysis were included in the multivariate analysis [21]. Statistical analyses were performed using R (version 4.1.2) [22]. 

## 3. Results

It was observed that 29 (5.43%) pregnant women were foreigners (27 Venezuelans, 1 Colombian, and 1 Peruvian). The majority of pregnant women were born in the Ecuadorian Sierra region (82.24%), were between 20 and 35 years old (75.33%), were of mixed race (89.53%), lived with their partners (83.93%), had 8 to 13 years of education (62.24%), had no income (62.24%), and had a family income of up to one basic salary equivalent (44.67%). More than half of the women were overweight (36.64%) or obese (14.21%). Only 19 pregnant women (3.55%) reported a personal medical history (gastritis: nine; allergic disorder: five; lactose intolerance: two; cholecystectomy: three; 60% visual deficit: one; and arterio-venous malformation: one) (Table 1).

Table 2 shows the food groups that were part of the dietary habits of the pregnant women.

The following three dietary patterns were identified (Table 3):

- The “dairy, salads, snacks, and sweet dressings” pattern, which contrasted with the consumption of sugary drinks (7% of the variance);
- The “refined carbohydrates” pattern (6% of the variance);
- The “traditional Ecuadorian” pattern, composed of animal protein, tubers, salted snacks and dressings, processed meats, and salads, contrasted with the consumption of soups (6% of the variance).

Pregnant women of foreign origin, those with an ethnic self-identification of “other”, and those born in the coastal and highland regions of Ecuador had a higher adherence to the “dairy, salads, and sweet snacks/dressings” dietary pattern. On the other hand, pregnant women with education levels equal to or less than 7 years and lower economic incomes showed a lower inclination towards the “refined carbohydrates” pattern. Additionally, it was found that the affinity for the “traditional Ecuadorian” pattern was higher among pregnant women born in the coastal region, those who had previously smoked tobacco, and those without a history of previous pregnancies (Table S1).

Table 4 displays the results of the bivariate analysis from which the variables were selected for multiple adjustments. For the “dairy, salads, and sweet snacks/dressings” pattern, the following variables were selected (*p* < 0.20): nationality, ethnicity, education level, unpaid work, cohabitation with a partner, the presence of a family history of metabolic diseases, and sedentary behaviour.

In the “refined carbohydrates” pattern, the variables (*p* < 0.20) selected for multiple adjustments were ethnicity, education level, income level, personal medical history, family history of metabolic diseases, previous alcohol consumption, and previous pregnancies.

The variables selected for multiple adjustments (*p* < 0.20) in the “traditional Ecuadorian” pattern were nationality, region, family history of metabolic diseases, previous tobacco smoking, and a history of previous pregnancies.

In the multivariate analysis (Table 5), a positive association was found between the “dairy, salads, snacks/sweet dressings” pattern and foreign nationality. The “refined carbohydrates” pattern showed a negative association with an education level equal to or less than 7 years and an income of up to USD 400 or one basic salary equivalent. The “traditional Ecuadorian” pattern showed a positive association with being born in the coastal region of Ecuador.

## 4. Discussion

In this study, three dietary patterns were identified among pregnant women living in Quito: the “dairy, salads, snacks/sweet dressings”, “refined carbohydrates”, and “traditional Ecuadorian” patterns. However, a positive association was only established between an affinity for the “dairy, salads, snacks/sweet dressings” pattern and foreign nationality. The “refined carbohydrates” pattern showed a negative association with an education level equal to or less than 7 years and an income of up to USD 400 (one basic salary equivalent), while the “traditional Ecuadorian” pattern was positively associated with being born in the coastal region of Ecuador. No associations were found between these patterns and conditions directly related to nutrition, such as overweight, obesity, low physical activity, and sedentary behaviour.

The analyses of the dietary patterns showed similarities with the patterns described in other studies, although these resemblances are not based on specific food groups but rather on the nature of the identified dietary patterns. One of the patterns is characterised by the presence of foods that cannot be classified into a typical dietary trend, while the other two are easily labelled as carbohydrate-rich patterns and traditional or country-specific patterns [21].

Studies have described the typical dietary patterns of pregnant women in their country or region, such as the traditional Brazilian and traditional Chinese patterns, which involve the consumption of common food groups in Brazil and China [21,23]. In the current study, a “traditional Ecuadorian” dietary pattern was identified, which included food groups specific to Ecuadorian culture. These findings suggest that dietary habits in each region are linked to the availability of locally produced foods [24]. 

The observations concerning the “dairy, salads, and sweet snacks/dressings” dietary pattern showed some similarity to the findings of a previous study in which a high daily consumption of dairy products, fruits, vegetables, and sugary beverages was observed in pregnant women from Spain [25]. On the other hand, studies that evaluated the dietary habits of pregnant Latin American women concluded that there is a low diversity of foods in their diets and that the intake of fruits and vegetables is insufficient in this population. In the present study, the consumption of vegetables (salad group) was representative of this dietary pattern, while fruits were excluded from the patterns due to their low communalities [25,26]. 

In a systematic review that analysed the diets of pregnant women from various nationalities, seven main food groups were identified, including carbohydrates, a finding consistent with the “refined carbohydrates” pattern in this study. Thus, it was confirmed that carbohydrates are part of the diets of many populations, regardless of their geographical locations [27].

In this study, it was also found that the “refined carbohydrates” pattern was inversely associated with incomes below USD 400 and with an education level equal to or less than 7 years. In this regard, it has been shown that high-risk dietary patterns (fried foods, desserts, and foods high in total fats) are associated with a higher socioeconomic status [28]. In another study, it was observed that pregnant women with fewer years of education had poorer-quality diets with high sugar consumption and a minimal intake of fruits, meats, and fibre [21]. In contrast, studies have indicated that pregnant women with higher educational levels show a higher consumption of healthy foods [29,30]. These results suggest an association among educational level, family income, and dietary pattern.

Women of foreign nationality showed a greater preference for the “dairy, salads, and sweet snacks/dressings” dietary pattern. In contrast, in another study of pregnant women in Spain, women of foreign nationality showed a positive association with the “healthy” dietary pattern that included vegetables, legumes, fish, meats, pasta, and rice. However, similar to the present study, the limited number of foreign women prevented a more detailed analysis of this group [31]. 

The results of this study suggest that the geographical locations and origins of pregnant women in Ecuador influence their food preferences. A positive association was found between an affinity for the “traditional Ecuadorian” dietary pattern and being born in the coastal region of Ecuador. This finding is interesting considering that the majority of the pregnant women in the study were from the highland region of the country. This result may be related to findings from another study in which it was observed that Ecuadorian immigrants in Spain maintained their traditional dietary habits, albeit in smaller quantities due to the limited availability and higher costs in the Spanish market [26]. This phenomenon may have led immigrants to adopt the local dietary patterns in Spain.

Regarding ethnicity, pregnant mestizo women showed a greater affinity for the “dairy, salads, snacks/sweet dressings” dietary pattern, while pregnant Afro-descendant women showed a lower affinity for this pattern. However, it is important to consider that the relationship between ethnicity and dietary patterns is complex and can vary depending on the geographical, cultural, and social contexts in which they occur. A previous study found that non-Hispanic Afro-descendant women followed a lower-quality diet in terms of nut, meat, whole fruit, legume, and cereal consumption [32]. 

No significant differences were found in the degree of affinity for the dietary patterns among pregnant women categorised according to whether they lived with a partner or not, as well as their employment statuses (paid or unpaid). However, another study found that women who have a partner are more likely to adhere to a healthy eating pattern than single women [33]. On the other hand, the association between employment and dietary patterns is contradictory. One study showed that women who were employed outside the home had a lower consumption of macronutrients and a lower calorie intake [34]. Conversely, it was established that employment was associated with a pattern rich in fast food and takeout, composed of “snacks, sandwiches, sweets, and soft drinks,” while unemployment was associated with the consumption of “sugary juices, bread and butter, rice and beans” [20]. These results highlight the complexity of the relationships among marital status, occupation status, and dietary pattern during pregnancy.

Although an association has been established between multiparity and the Western dietary pattern composed of refined grains, fats, potatoes, sweets, and processed meats [35], no association was found between previous pregnancies and an affinity for the dietary patterns in this study. Multiparity may be a confounding factor concerning sociodemographic variables, such as age, marital status, and family income. Consequently, these findings are considered inconsistent [36].

Although 50% of the pregnant women had a BMI in the overweight (36.64%) and obese (14.21%) ranges, no significant differences were found in terms of affinity for the dietary patterns. These results are consistent with the findings reported in another study that also did not find a relationship between dietary patterns and overweight or obesity in the general population. However, the cross-sectional nature of this study may have underestimated the food consumption of individuals with overweight or obesity [31]. Another study found that pregnant women with a high BMI tend to consume diets that are low in vegetables, fruits, meats, dairy, and grains [37]. Therefore, further research is needed to better understand the relationship between BMI and dietary patterns in pregnant women.

In the current study, no relationship was found between the degree of affinity for the dietary patterns and previous or current alcohol consumption in pregnant women. However, it has been observed that women tend to significantly decrease their alcohol intake during pregnancy [38]. A previous study found that consuming more than one standard unit of alcohol per day during the first trimester was associated with a higher adherence to the “processed” dietary pattern, characterised by a high consumption of processed meats and a low consumption of fruits and vegetables, while lower alcohol consumption was associated with the “health-conscious” pattern, characterised by a high consumption of fruits, vegetables, and whole grains [36]. No association was found between tobacco smoking or exposure to tobacco smoke and affinity for the dietary patterns in pregnant women in the city of Quito. However, it has been reported that pregnant women who smoke consume fewer whole grains and fruits but consume more legumes and sugary beverages than nonsmokers [4]. More studies are needed to determine if alcohol consumption and tobacco smoking influence the dietary patterns of pregnant women.

When analysing different levels of physical activity and affinity towards the three dietary patterns, no significant associations were found. However, previous studies have shown that there is an association between physical activity during pregnancy and the adoption of healthy eating habits [35,39]. 

In the current study, no association was found between the affinity for the dietary patterns and a personal or family history of metabolic diseases in pregnant women. In contrast, findings from another study suggested that women without a history of diseases were 1.7 times more likely to maintain good dietary practices during pregnancy, including consuming adequate amounts of fruits, vegetables, and proteins [40]. Additionally, it was observed that the affinity for the “sugar-sweetened juices, bread and butter, rice and beans” pattern in pregnant women was positively associated with the absence of a family history of hypertension [20]. 

Although a direct comparison of the diets of pregnant women in Quito with other similar studies in Ecuador could not be made, a study conducted at Hospital General Babahoyo revealed a concerning lack of consumption of healthy foods among pregnant women. In the mentioned study, 68% of women did not consume fruits, 46% did not eat legumes, 5% did not consume vegetables, and only 7% met the minimum dairy intake recommendation. Additionally, 75% of the women consumed snacks, and none of them engaged in physical activity during their pregnancies [41]. These findings highlight the need to promote healthy eating and an active lifestyle during pregnancy throughout the country.

Similar to studies conducted in other populations of pregnant women, the results regarding the influence of sociodemographic and lifestyle factors on the affinity for the dietary patterns in this research are diverse. Therefore, dietary patterns are influenced by cultural, social, and personal factors, as well as by individual preferences. It has been observed that women perceive pregnancy as an opportunity to relax their previously established dietary and physical activity restrictions, which can lead to the consumption of unhealthy foods and a sedentary lifestyle during this period [42]. On the other hand, some studies did not find significant differences in the diets of women during pregnancy compared to their pre-pregnancy food intake habits [29]. These findings, including those presented in this research, emphasise the need to plan and implement interventions at the community, family, and individual levels to improve the affinity for the consumption of healthy foods.

Finally, it is important to note that, in this study, pregnant women were recruited from institutions that are part of Ecuador’s public health system. This leads to the conclusion that the study was conducted with pregnant women who represent a population with a lower socioeconomic status, lower levels of education, and/or elementary occupations. Further research is needed to identify both the dietary patterns and the relationship among sociodemographic factors, lifestyle factors, and dietary patterns in pregnant women in Ecuador.

One weakness of this study is that the diets of the pregnant women could have been underestimated, as reported in other studies investigating food intake in pregnant women. Additionally, a limitation of the cross-sectional design is that it only provides information from a specific moment in time, which means that if the same population is examined at another time, the results may vary [43].

This study provides an overview of the dietary patterns in pregnant women from the city of Quito and Ecuador as a whole, as the Hospital Gineco Obstétrico Isidro Ayora is a national reference hospital. Additionally, the use of 24 h dietary recalls is a strength, as they were conducted after food consumption, enhancing the participants’ ability to recall most of the foods they consumed as part of their diets and reducing the likelihood of interference with dietary behaviour [17,18]. 

## 5. Conclusions

The dietary patterns of pregnant women are affected by various sociodemographic and lifestyle factors. This study offers an overview of the dietary patterns of pregnant women in Quito, highlighting the influence of sociodemographic factors on dietary preferences during pregnancy. When comparing these results with previous research, the influence of geographical and cultural diversity on these dietary patterns is evident.

It is crucial to carry out more studies that deepen the understanding of these dietary patterns and analyse their nutritional and caloric properties. This will allow for the development of more effective policies to promote healthy eating habits and lifestyles during pregnancy.

## Figures and Tables

**Table 1:** Distribution of categorical sociodemographic and lifestyle variables.

Categories	Frequency, *n* (%)
Nationality	
Ecuadorian	506 (94.58)
Foreign	29 (5.45)
Region of origin in Ecuador (*n* = 506)	
The highlands (Sierra)	440 (86.95)
Coast (Costa)	55 (10.86)
Eastern region (Oriente)	10 (1.97)
Galápagos Islands (Insular)	1 (0.19)
Age	
<20 years	50 (9.35)
20–35 years	403 (75.32)
>35 years	82 (15.33)
Lives with a partner	
Yes	449 (83.93)
No	86 (16.07)
Ethnicity	
Mestiza	479 (89.53)
Indigenous	29 (5.42)
Afro-descendant	16 (2.99)
Other	11 (2.06)
Years of education	
≤7 years	39 (7.29)
8–13 years	333 (62.24)
≥14 years	163 (30.47)
Occupation type	
With remuneration	202 (37.76)
Without remuneration	333 (62.24)
Family income	
<USD 400	239 (44.67)
USD 400–800	225 (42.06)
USD 800–1200	46 (8.60)
>USD 1200	25 (4.67)
Personal medical history	
Yes	19 (3.55)
No	516 (96.45)
Family history of metabolic diseases	
Yes	221 (41.31)
No	314 (58.69)
Previous alcohol use	
Yes	77 (14.39)
No	458 (85.61)
Current consumption of alcohol	
Yes	1 (0.19)
No	534 (99.81)
Previous tobacco use	
Yes	24 (4.49)
No	511 (95.51)
Current tobacco use	
Yes	1 (0.19)
No	534 (99.81)
Current exposure to tobacco smoke	
Yes	29 (5.42)
No	506 (94.58)
Physical activity	
Active	256 (47.85)
Inactive	279 (52.15)
Sedentary behaviour	
<2 h	170 (31.78)
2–4 h	215 (40.19)
4–6 h	113 (21.12)
>6 h	37 (6.92)
Previous pregnancies	
Yes	336 (62.80)
No	199 (37.20)
Number of previous pregnancies, (*n* = 336)	
1–3	312 (92.85)
>4	24 (7.14)
BMI for gestational age at initial assessment	
Low (<18.5)	8 (1.50)
Normal (18.5–24.9)	255 (47.66)
Overweight (25–29.9)	196 (36.64)
Obesity (≥30)	76 (14.21)

BMI = body mass index.

**Table 2:** Grouping of foods included in the 24 h dietary recalls.

Food Group	Included Foods
Flours	Breads, doughnuts, wind empanadas, toast, tortillas/pancakes/arepas, pastries, humitas (corn-based dish), tamales, quimbolitos (Ecuadorian sweet corn cake), pristiños (Ecuadorian fried dough), sponge cakes, pizza.
Green, ripe bananas	Empanadas, bolón/tortillas, mashed/cooked green plantains, plantain chips, tostones, fried ripe plantains, cooked ripe plantains.
Eggs	Cooked egg, fried/scrambled egg, omelette.
Dairy products	Cheese, whole milk, skim milk, chocolate milk, milk coffee, fruit smoothie, colostrum, cream, yogurt.
Fruits	Apple, banana, plantain, watermelon, cantaloupe, grape, pear, mango, sapodilla, kiwi, cherry, starfruit, papaya, pineapple, orange, strawberry, blackberry, custard apple, prickly pear, peach, passion fruit, mandarin, capulin, dragon fruit, plum, currant, fig, fruit salad, fruit juice.
Soups	Noodle soup, minestrone soup, vegetable soup, cow foot soup, chicken broth, green plantain dumpling soup, quinoa soup, hominy soup, locro (Ecuadorian potato soup), oatmeal soup, wheat soup, barley soup, lentil soup, rib broth, cream soup, morocho soup, fish soup, encebollado (Ecuadorian fish soup), sancocho (Latin American meat and vegetable stew).
Rice and noodles	White rice, stuffed rice/chaulafán (a traditional Ecuadorian dish with rice and various fillings), noodles.
Grains	Cooked beans, bean stew, lentil stew, corn, peas, fava beans, chickpeas, lupini beans, hominy, salted/sweet toasted corn, popcorn, oat grains.
Animal protein sources	Cooked beef, fried/breaded beef, ground beef, fried chicken, cooked/baked chicken, turkey, fried pork, cooked pork, pork rinds, pork skin, lamb, guinea pig, liver, other offal, fried fish, cooked fish, clams, shrimp, sushi, sardines, tuna, ceviche.
Cured meats	Sausage, ham, chorizo, bologna.
Salads	Tomato, green/purple cabbage, onion, lettuce, cooked carrot, raw carrot, bell pepper, green bean, radish, cucumber, zucchini, broccoli/cauliflower, beetroot, avocado, mushroom, olive, spinach, Russian salad.
Tubers	French fries, boiled potatoes, mashed potatoes, potato omelettes, melloco (a type of Andean tuber), yucas (cassava).
Sugary beverages	Colada (a traditional Latin American hot beverage made with milk and coffee), chocolate in water, flavoured water, artificial juice, juice with sugar, oat milk, chicha (a fermented corn beverage), malt beverage, coffee with water, soda.
Savoury snacks and dressings	Tomato sauce, mayonnaise, BBQ sauce, hot dogs, hamburgers, tacos, lasagna, Tostitos/Doritos (corn chips), potato chips, crackers, k-chitos (corn snacks), pork rinds, chili sauce, margarine.
Sweet snacks and dressings	Chocolate, ice cream, jelly, marshmallow fluff, candy/lollipop/gummy candies, cookies, cereal/rice crispy treats, granola, jam, coconut candy, Nutella/hazelnut spread, dulce de leche, whipped cream, caramel sauce, condensed milk, honey.
Nuts and dried fruits	Walnuts, almonds, peanuts, blueberries, raisins.

**Table 3:** Factor loading distributions or correlation coefficients of food groups within the dietary patterns.

Food Groups	Dairy, Salads, and Sweet Snacks/Dressings	Refined Carbohydrates	Traditional Ecuadorian
Dairy products	0.71	0.07	−0.06
Sugary beverages	−0.50	0.04	<0.01
Salads	0.28	−0.04	0.27
Sweet snacks and dressings	0.25	0.07	0.16
Nuts and dried fruits	0.20	−0.04	0.03
Flours	<0.01	0.82	0.02
Fruits	0.10	−0.14	0.04
Soups	−0.19	−0.18	−0.51
Animal protein sources	<0.01	−0.21	0.38
Tubers	0.05	−0.02	0.32
Savoury snacks and dressings	0.04	−0.05	0.28
Cured meats	0.05	0.02	0.25
Green, ripe bananas	−0.04	−0.19	0.22
Rice and noodles	−0.05	−0.11	0.18
Variance (%)	7	6	6
Cumulative variance (%)	7	13	19

Notes: The food groups “eggs” and “grains” were excluded from the analysis due to their low communalities. Factorial analysis performed with extraction by principal components.

**Table 4:** Bivariate analysis of sociodemographic and lifestyle characteristics of pregnant women and their eating patterns.

Nutritional Pattern	Dairy, Salads, and Sweet Snacks/Dressings			Refined Carbohydrates			Traditional Ecuadorian		
Variable	β	95% CI	*p*	β	95% CI	*p*	β	95% CI	*p*
Foreign nationality	1.01	0.64; 1.37	<0.01	−0.28	−0.75; 0.18	0.23	0.38	−0.14; 0.91	0.15
Coast region (*n* = 506)	−0.05	−0.33; 0.22	0.69	−0.13	−0.48; 0.21	0.45	0.62	0.22; 1.01	<0.01
Age > 35 years	−0.01	−0.25; 0.25	0.90	0.02	−0.27; 0.31	0.89	−0.18	−0.51; 0.14	0.26
Mestizo ethnicity	−0.32	−0.60; −0.04	0.02	0.44	0.09; 0.79	0.01	−0.10	−0.49; 0.28	0.60
Education level ≤ 7 years	−0.28	−0.61; 0.04	0.08	−0.76	−1.16; −0.35	<0.01	−0.07	−0.53; 0.38	0.74
Work without pay	−0.12	−0.29; 0.05	0.17	−0.01	−0.29; 0.26	0.90	0.14	−0.16; 0.45	0.37
Not living with a partner	0.37	0.15; 0.59	<0.01	−0.09	−0.38; 0.20	0.54	−0.13	−0.45; 0.19	0.42
Income up to USD 400	−0.03	−0.20; 0.13	0.66	−0.40	−0.61; −0.18	<0.01	−0.08	−0.03; 0.16	0.51
Personal medical history	0.02	−0.43; 0.48	0.92	−0.44	−1.01; 0.13	0.13	0.40	−0.23; 1.05	0.21
Family history of metabolic diseases	0.16	−0.00; 0.33	0.06	−0.20	−0.42; 0.00	0.06	0.18	−0.05; 0.42	0.13
Previous tobacco use	−0.07	−0.48; 0.34	0.73	0.26	−0.25; 0.78	0.31	0.48	−0.09; 1.05	0.10
Current tobacco use	1.19	−0.77; 3.17	0.23	−0.39	−2.87; 2.07	0.75	−0.75	−3.52; 2.01	0.59
Exposed to tobacco smoke	−0.00	−0.38; 0.36	0.96	−0.09	−0.57; 0.37	0.67	−0.14	−0.67; 0.37	0.58
Previous consumption of alcohol	−0.09	−0.33; 0.14	0.44	−0.23	−0.53; 0.07	0.13	0.05	0.28; 0.39	0.74
Current alcohol use	−0.26	−2.24; 1.71	0.79	−0.94	−3.42; 1.52	0.45	−1.14	−3.91; 1.62	0.41
Elevated BMI for gestational age	−0.04	−0.21; 0.12	0.58	0.020	−0.19; 0.23	0.85	0.06	−0.17; 0.30	0.59
History of previous pregnancies	0.00	−0.17; 0.18	0.94	−0.03	−0.25; 0.18	0.77	−0.22	−0.47; 0.12	0.07
4 or more previous gestations (*n* = 336)	−0.17	−0.58; 0.24	0.40	−0.43	−0.95; 0.09	0.10	−0.06	−0.66; 0.53	0.83
Physically active	−0.01	−0.18; 0.15	0.88	−0.07	−0.29; 0.13	0.46	−0.14	−0.37; 0.09	0.25
Sedentary behaviour > 4 h	0.21	0.02; 0.40	0.02	−0.00	−0.23; 0.23	0.99	0.00	−0.26; 0.27	0.97

Method: linear regression.

**Table 5:** Multivariate analysis of sociodemographic and lifestyle characteristics of pregnant women and their dietary patterns.

Variable	β	95% CI	*p*
Dairy, salads, and sweet snacks/dressings			
Foreign nationality	0.82	0.43; 1.21	<0.01
Mestizo ethnicity	−0.13	−0.41; 0.15	0.36
Education level ≤ 7 years	−0.20	−0.53; 0.11	0.20
Work without pay	−0.07	−0.24; 0.09	0.08
Not living with a partner	0.20	−0.02; 0.42	0.07
Family history of metabolic diseases	0.13	−0.03; 0.30	0.11
Sedentary behaviour > 4 h	−0.15	−0.03; 0.33	0.10
Refined carbohydrates			
Mestizo ethnicity	0.23	−0.18; 0.65	0.28
Education level ≤ 7 years	−0.59	−1.05; −0.14	<0.01
Income up to USD 400	−0.52	−0.79; −0.25	<0.01
Personal medical history	−0.47	−1.09; 0.13	0.12
Family history of metabolic diseases	0.04	−0.22; 0.30	0.75
Previous consumption of alcohol	−0.14	−0.53; 0.23	0.45
4 or more previous gestations	−0.14	−0.66; 0.37	0.58
Traditional Ecuadorian			
Foreign nationality	0.46	−0.06; 0.98	0.08
Coast region	0.62	0.22; 1.01	<0.01
Family history of metabolic diseases	0.12	−0.11; 0.36	0.31
Previous tobacco use	0.49	−0.07; 1.06	0.09
History of previous pregnancies	−0.22	−0.46; 0.02	0.07

Method: multiple linear regression.

## Data Availability

Data are available only upon request from the corresponding author due to ethical restrictions under Ecuadorian regulations.

## Supplementary Materials

The following supporting information can be downloaded at https://www.mdpi.com/article/10.3390/nu16040475/s1, Table S1. Degree of affinity with nutritional patterns by sociodemographic and lifestyle factors in Quito in 2023 (*n* = 535).
