# Supplementary material for: Dietary Patterns and Factors Associated with Food Affinity in Pregnant Women from Quito, Ecuador
Source: Nutrients. 2024 Feb 7;16(4):475. doi: 10.3390/nu16040475 (PMC10892012; doi:10.3390/nu16040475)
Supplement: Supplementary file 1 [file nutrients-16-00475-s001.zip › nutrients-2817680-supplementary.pdf]

Table S1. Degree of affinity with nutritional patterns by sociodemographic and lifestyle factors in Quito in 2023 (n = 535)

Variables	N	Dairy, salads, and sweet snacks/dressings				Refined carbohydrates				Traditional Ecuadorian			
		Low affinity n (%)	Medium affinity n (%)	High affinity n (%)	p	Low affinity n (%)	Medium affinity n (%)	High affinity n (%)	p	Low affinity n (%)	Medium affinity n (%)	High affinity n (%)	p
Nationality													
Ecuadorian	506	276 (54.5)	22 (43.9)	8 (1.6)	<0.01	184 (36.4)	260 (51.4)	62 (12.3)	0.65	79 (15.6)	347 (68.6)	80 (15.8)	0.39
Foreign	29	8 (27.6)	18 (62.1)	3 (10.3) $^{+}$		12 (41.4)	15 (51.7)	2 (6.9)		2 (6.9)	21 (72.4)	6 (20.7)	
Region of origin in Ecuador (n = 506)													
The highlands (Sierra)	440	242 (55)	191 (43.4)	7 (1.6) $^{+}$	<0.01	157 (35.7)	277 (51.6)	56 (12.7)	0.48	74 (16.8)	301 (68.4)	65 (14.8)	0.04
Coast (costa)	55	29 (52.7)	25 (45.5)	1 (1.8) $^{+}$		25 (45.5)	24 (43.6)	6 (10.9)		3 (5.5)	38 (69.1)	14 (25.5) $^{+}$	
Eastern region (Oriente)	10	4 (40)	6 (60)	0 (0)		2 (20)	8 (80)	0 (0)		1 (10)	8 (80)	1 (10)	
Galápagos Islands (Insular)	1	1 (100)	0 (0)	0 (0)		0 (0)	1 (100)	0 (0)		1 (100)	0 (0)	0 (0)	
Age													
< 20 years	50	29 (58)	21 (42)	0 (0)	0.65	20 (40)	22 (44)	8 (16)	0.78	10 (20)	31 (62)	9 (18)	0.81
20-35 years	403	214 (53.1)	179 (44.4)	10 (2.5)		148 (36.7)	209 (51.9)	46 (11.4)		58 (14.4)	280 (69.5)	65 (16.1)	
> 35 years	82	41 (50)	40 (48.8)	1 (1.2)		28 (34.1)	44 (53.7)	10 (12.2)		13 (15.9)	57 (69.5)	12 (14.6)	
Lives with a partner													
Yes	449	246 (54.8)	194 (43.2)	9 (2)	0.19	166 (37)	231 (51.4)	52 (11.6)	0.80	67 (14.9)	313 (69.7)	69 (15.4)	0.52
No	86	38 (44.2)	46 (53.5)	2 (2.3)		30 (34.9)	44 (51.2)	12 (14)		14 (16.3)	64 (55)	17 (19.8)	
Ethnicity													
Mestiza	479	260 (54.3)	211 (44.1)	8 (1.7)	<0.01	171 (35.7)	248 (51.8)	60 (12.5)	0.53	69 (14.4)	333 (69.5)	77 (16.1)	0.82

Indigenous	29	12 (41.4)	16 (55.2)	1 (3.4)		12 (41.4)	15 (51.7)	2 (6.9)		6 (20.7)	18 (62.1)	5 (17.2)	
Afro-descendant	16	9 (56.2)	7 (43.8)	0 (0)		6 (37.5)	9 (56.2)	1 (6.2)		4 (25)	9 (56.2)	3 (18.8)	
Other	11	3 (27.3)	6 (54.5)	2 (18.2) [+]		7 (63.6)	3 (27.3)	1 (9.1)		2 (18.2)	8 (72.7)	1 (9.1)	
Years of education													
≤ 7 years	39	26 (66.7)	13 (33.3)	0 (0)	0.06	26 (66.7) [++]	9 (23.1)	4 (10.3)	<0.01	11 (28.2)	23 (59)	5 (12.8)	0.16
8-13 years	333	177 (53.2)	152 (45.6)	4 (1.2)		115 (34.5)	180 (54.1)	38 (11.4)		49 (14.7)	233 (70)	51 (15.3)	
≥ 14 years	163	81 (49.7)	75 (46)	7 (4.3)		55 (33.7)	86 (52.8)	22 (13.5)		21 (12.9)	112 (68.7)	30 (18.4)	
Occupation type													
With remuneration	202	100 (49.5)	95 (47)	7 (3.5)	0.12	63 (31.2)	114 (56.4)	25 (12.4)	0.11	28 (13.9)	140 (69.3)	34 (16.8)	0.76
Without remuneration	303	184 (55.3)	145 (43.5)	4 (1.2)		133 (39.9)	161 (48.3)	39 (11.7)		53 (15.9)	228 (68.5)	52 (15.6)	
Family income													
< 400 USD	241	138 (57.3)	98 (40.7)	5 (2.1)	0.23	107 (44.4) [++]	112 (46.5)	22 (9.1)	0.02	43 (17.8)	163 (67.6)	35 (14.5)	0.27
400-800 USD	223	111 (49.8)	109 (48.9)	3 (1.3)		68 (30.5)	122 (54.7)	33 (14.8)		32 (14.3)	155 (69.5)	36 (16.1)	
800-1200 USD	45	23 (51.1)	21 (46.7)	1 (2.2)		15 (33.3)	26 (57.8)	4 (8.9)		5 (11.1)	33 (73.3)	7 (15.6)	
> 1200 USD	26	12 (46.2)	12 (46.2)	2 (7.7)		6 (23.1)	15 (57.7)	5 (19.2)		1 (3.8)	17 (65.4)	8 (30.8)	
Personal medical history													
Yes	19	10 (52.6)	9 (47.4)	0 (0)	0.8	11 (57.9)	7 (36.8)	7 (5.3)	0.13	1 (5.3)	354 (73.3)	4 (21.1)	0.44
No	516	274 (52.6)	231 (47.4)	11 (2.1)		185 (35.9)	268 (51.9)	63 (12.2)		80 (15.5)	354 (68.6)	82 (15.9)	
Family history of metabolic diseases													

Yes	221	111 (50.2)	103 (43.6)	7 (3.2)	0.20	93 (42.1)	105 (47.5)	23 (10.4)	0.08	28 (12.7)	153 (69.2)	40 (18.1)	0.29
No	314	173 (55.1)	137 (43.6)	4 (1.3)		103 (32.8)	170 (54.1)	41 (13.1)		53 (16.9)	215 (68.5)	46 (14.6)	
Previous alcohol use													
Yes	77	43 (55.8)	33 (42.9)	1 (1.3)	0.90	31 (40.3)	39 (50.6)	7 (9.1)	0.62	10 (13)	52 (67.5)	15 (19.5)	0.61
No	458	241 (52.6)	207 (45.2)	10 (2.2)		165 (36)	236 (51.5)	57 (12.4)		71 (15.5)	316 (69)	71 (15.5)	
Previous tobacco use													
Yes	24	14 (58.3)	9 (37.5)	1 (4.2)	0.61	6 (25)	14 (58.3)	4 (16.7)	0.44	3 (12.5)	12 (50)	9 (37.5) $^{+}$	0.01
No	511	270 (52.8)	231 (45.2)	10 (2)		190 (37.2)	261 (51.1)	60 (11.7)		78 (15.3)	356 (69.7)	77 (15.1)	
Current exposure to tobacco smoke													
Yes	29	16 (55.2)	12 (41.4)	1 (3.4)	0.81	11 (37.9)	15 (51.7)	3 (10.3)	0.95	5 (17.2)	19 (65.5)	5 (17.2)	0.92
No	506	268 (53)	228 (41.4)	10 (2)		185 (36.6)	260 (51.4)	61 (12.1)		76 (15)	349 (69)	81 (16)	
Physical activity													
Active	256	140 (54.7)	112 (43.8)	4 (1.6)	0.62	97 (37.9)	130 (50.8)	29 (11.3)	0.81	40 (15.6)	180 (70.3)	36 (14.1)	0.47
Inactive	279	144 (51.6)	128 (45.9)	7 (2.5)		99 (35.5)	145 (52)	35 (12.5)		41 (14.7)	188 (67.4)	50 (17.9)	
Sedentary behaviour													
< 4 hours	385	211 (54.8)	169 (43.9)	5 (1.3)	0.08	141 (36.6)	199 (51.7)	45 (11.7)	0.94	55 (14.3)	264 (68.6)	66 (17.1)	0.43
> 4 hours	150	73 (48.7)	71 (47.3)	6 (4)		55 (36.7)	76 (50.7)	19 (12.7)		26 (17.3)	104 (69.3)	20 (13.3)	
Previous pregnancies													
Yes	336	176 (52.4)	153 (45.5)	7 (2.1)	0.91	120 (35.7)	177 (52.7)	39 (11.6)	0.74	61 (18.2)	225 (67)	50 (14.9)	0.03

No	199	108 (54.3)	87 (43.7)	4 (2)		76 (38.2)	98 (49.2)	25 (12.6)		20 (10.1)	143 (71.9)	36 (18.1)$^{+}$	
Number of previous pregnancies (n = 336)													
1-3	312	163 (52.2)	142 (45.5)	7 (2.2)	0.75	108 (34.6)	168 (53.8)	36 (11.5)	0.26	55 (17.6)	212 (67.9)	45 (14.4)	0.38
≥ 4	24	13 (54.2)	11 (45.8)	0 (0)		12 (50)	9 (37.5)	9 (12.5)		6 (25)	6 (54.2)	13 (20.8)	
BMI for gestational age at initial assessment													
Low	8	6 (75)	1 (12.5)	1 (12.5)	0.22	5 (62.5)	1 (12.5)	2 (25)	0.31	2 (25)	4 (50)	2 (25)	0.64
Normal	255	131 (51.4)	120 (47.1)	4 (1.6)		95 (37.3)	130 (51)	30 (11.8)		40 (15.7)	177 (69.4)	38 (14.9)	
Overweight	196	106 (54.1)	85 (43.4)	5 (2.6)		66 (33.7)	104 (53.1)	26 (13.3)		25 (12.8)	140 (71.4)	31 (15.8)	
Obesity	76	41 (53.9)	34 (44.7)	1 (1.3)		30 (39.5)	40 (52.6)	6 (7.9)		14 (18.4)	47 (61.8)	15 (19.7)	

$^{+}$, High-affinity group

<, less than

>, greater than

≤, less than or equal to

$^{++}$, Low-affinity group

≥, greater than or equal to

USD, US dollars.

Method: Test Chi-square.
